# Environmental Photocatalytic Degradation of Antidepressants with Solar Radiation: Kinetics, Mineralization, and Toxicity

**DOI:** 10.3390/nano11030632

**Published:** 2021-03-03

**Authors:** Nina Finčur, Daniela Šojić Merkulov, Predrag Putnik, Vesna Despotović, Nemanja Banić, Marina Lazarević, Dragana Četojević-Simin, Jasmina Agbaba, Biljana Abramović

**Affiliations:** 1 Department of Chemistry, Biochemistry and Environmental Protection, University of Novi Sad Faculty of Sciences, Trg Dositeja Obradovića 3, 21000 Novi Sad, Serbia; nina.fincur@dh.uns.ac.rs (N.F.); vesna.despotovic@dh.uns.ac.rs (V.D.); nemanja.banic@dh.uns.ac.rs (N.B.); marina.lazarevic@dh.uns.ac.rs (M.L.); jasmina.agbaba@dh.uns.ac.rs (J.A.); biljana.abramovic@dh.uns.ac.rs (B.A.); 2 Department of Food Technology, University North, Trg dr. Žarka Dolinara 1, 48000 Koprivnica, Croatia; 3 Oncology Institute of Vojvodina, Dr Goldmana 4, 21204 Sremska Kamenica, Serbia; ddaaggeerr@gmail.com

**Keywords:** amitriptyline, photocatalysis, kinetics, mineralization, toxicity assessment

## Abstract

This work is focused on the kinetics, mineralization, and toxicological assessments of the antidepressant drug amitriptyline hydrochloride (AMI) in UV or solar illuminated aqueous suspensions of ZnO, TiO_2_ Degussa P25, and TiO_2_ Hombikat. ZnO was proven to be the most effective photocatalyst, and it was used for all further experiments under solar irradiation. The highest reaction rate was observed at 1.0 mg/mL of catalyst loading. In the investigated initial concentration range (0.0075–0.3000 mmol/L), the degradation rate of AMI increased with the increase of initial concentration in the investigated range. The effects of H_2_O_2_, (NH_4_)_2_S_2_O_8_, and KBrO_3_, acting as electron acceptors, along with molecular oxygen were also studied. By studying the effects of ethanol and NaI as a hydroxyl radical and hole scavenger, respectively, it was shown that the heterogeneous catalysis takes place mainly via free hydroxyl radicals. In the mineralization study, AMI photocatalytic degradation resulted in ~30% of total organic carbon (TOC) decrease after 240 min of irradiation; acetate and formate were produced as the organic intermediates; NH_4_^+^, NO_3_^−^, NO_2_^−^ ions were detected as nitrogen byproducts. Toxicity assessment using different mammalian cell lines, showed that H-4-II-E was the most sensitive one.

## 1. Introduction

Nowadays, pharmaceutically active compounds (PACs) represent a group of chemicals that are used in large quantities throughout the world. After use, PACs are excreted unchanged or as metabolites in the urine and feces, and enter the wastewater treatment plants from which some of the components are practically unmodified and discharged into the environment [1,2]. In addition, PACs can reach the environment due to improper waste disposal of unused or expired drugs [3]. Their persistence in aquatic systems can lead to a potential risk for living organisms. Among the various types of conventional methods for the purification of water, advanced oxidation processes (AOPs) have proven to be very effective for the removal of PACs from aquatic environments [4]. AOPs are defined as processes that generate highly reactive species, mostly hydroxyl radicals (^•^OH radicals), which can mineralize a large number of harmful substances [5].

In recent years, a number of studies in the field of heterogeneous photocatalysis have been conducted due to the high efficiency of the degradation and mineralization of organic compounds, but also because of possible uses in the visible and UV range [6]. In most cases, heterogeneous photocatalysis is related to semiconductor photocatalysis, which can be used for the removal of organic and inorganic pollutants from the water and the gas phase systems. The most commonly used photocatalysts are TiO_2_ and ZnO, which have a high photochemical stability, photosensitivity, non-toxic nature, and strong oxidation power [7]. Their electronic structure causes photocatalysts to act as sensitizers for redox processes induced by light [8]. Semiconductor photocatalysis is the result of the interaction of electron−hole pairs (e^−^−h^+^), which occur after irradiation of semiconductors in the presence of oxygen and hydroxyl ions, producing highly reactive radicals [9]. These e^−^−h^+^ pairs can either recombine and release the absorbed energy in the form of heat or participate in oxidation–reduction reactions that can lead to the degradation of pollutants [10]. Ahmed et al. [6] showed that the efficiency of photocatalytic degradation depends on the type of catalyst, pH, type of organic substrate and its concentration, intensity and type of irradiation, ionic composition of wastewater, etc. Understanding the impacts of various factors on the photocatalytic efficiency is of significant importance for the design and selection of effective techniques for the purification of different wastewater [6].

Depression and anxiety are common forms of mental illness. Amitriptyline hydrochloride (AMI; 3-(10,11-dihydro-5H-dibenzo[a,d][7]annulen-5-ylidene)-N,N-dimethylpropan-1-amine hydrochloride, CAS No. 549-18-8) is the most widely used tricyclic antidepressant. It is commonly used for this in clinical treatments, but it is also used against acute headache, insomnia, migraines, etc. [11,12]. AMI is found in drinking water in France at a concentration of 1.4 ng/L [13], as well as in surface waters in the UK at a concentration of 0.5 to 21 ng/L [14]. In addition, AMI was also found in the solid residues from the treatment of wastewater in Canada at a concentration of 448 ng/g [15].

In this paper, the efficiency of various semiconductor photocatalysts (ZnO, TiO_2_ Degussa P25 (TiO_2_-D), and TiO_2_ Hombikat (TiO_2_-H)) were compared for the photocatalytic degradation of an aqueous suspension of AMI. After the selection of the most active photocatalyst, subsequent experiments were performed in order to investigate the influence of different process parameters (irradiation type, UV and simulated solar (solar) radiation, photocatalyst loading, and the initial AMI concentration) on the process performance. In addition, the effects of electron acceptors and the presence of radical/hole scavengers were investigated. To study the kinetics of the photocatalytic reaction, liquid chromatography was used, while mineralization degree was evaluated by using total organic carbon analysis and ion chromatography measurements. In order to evaluate the influence of the waters containing AMI and possible intermediates formed during photocatalytic degradation on the environment, toxicity assessment using rat hepatoma (H-4-II-E), mouse neuroblastoma (Neuro-2a), human colon adenocarcinoma (HT-29), and human fetal lung (MRC-5) mammalian cell lines were performed in vitro.

## 2. Materials and Methods

### 2.1. Materials and Reagents

**Figure 1:**
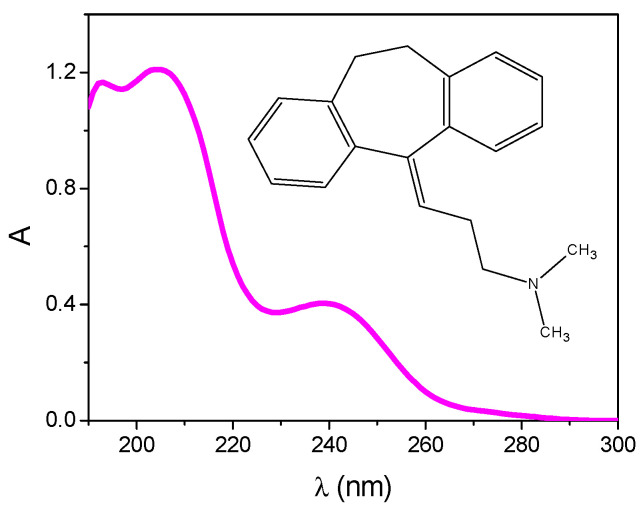
Absorption spectrum of amitriptyline hydrochloride (AMI) aqueous solution (0.0300 mmol/L). Inset represents structural formula of AMI.

AMI (≥98%, Sigma–Aldrich, Chemie GmbH, Munich, Germany), the absorption spectrum and structure of which are shown in Figure 1 and major properties of which are summarized in Table 1, was used without further purification. Acetonitrile (99.9%, Sigma−Aldrich, St. Louis, MO, USA) and orthophosphoric acid (85%, pro analysis, Sigma–Aldrich, St. Louis, MO, USA) were used as the components of the mobile phase for liquid chromatography.

ZnO (99.9%, Sigma–Aldrich, crystallite size of 41.0 ± 0.9 nm, specific pore volume of 0.016 cm^3^/g, and specific surface area of 6.5 m^2^/g) [17], TiO_2_-D (75% anatase and 25% rutile, with average particle size from about 20 nm, according to the producer’s specification, total pore volume 0.134 cm^3^/g, and specific surface area of 53.2 m^2^/g) [18], and TiO_2_-H (anatase, Sigma–Aldrich, specific surface area of 35–65 m^2^/g) were used as photocatalysts.

Ethanol (pro analysis, Sigma–Aldrich, Chemie GmbH, Munich, Germany) and NaI (Carlo Erba, Reagents, Val de Reuil, France) were used as scavengers of ^•^OH radicals. Moreover, H_2_O_2_ (30%, Sigma–Aldrich, Chemie GmbH, Munich, Germany), KBrO_3_, and (NH_4_)_2_S_2_O_8_ (Merck, Darmstadt, Germany) were used as electron acceptors. Investigated cell lines were H-4-II-E (ATCC CRL-1548), HT-29 (ECACC 91072201), MRC-5 (ECACC 05090501), and Neuro-2a (ATCC CCL-131). Dulbecco’s modified Eagle’s medium (DMEM) and fetal calf serum purchased from PAA Laboratories GmbH (Pashing, Austria), penicillin and streptomycin from Galenika (Belgrade, Serbia), trypsin from Serva (Heidelberg, Germany), EDTA and trichloroacetic acid (TCA) from Laphoma (Skopje, FYR Macedonia), and tris(hydroxymethyl)amino methane from Sigma–Aldrich (St. Louis, MO, USA) were used for toxicity assessment.

### 2.2. Photodegradation Procedure

Photodegradation experiments were performed in the photochemical cell described previously by our group [19] and operational variables applied during the photocatalytic degradation experiments were shown in Table 2. Experiment of direct photolysis was performed under the same conditions as heterogeneous photocatalytic degradation, but without the addition of a photocatalyst. In the investigation of the influence of electron acceptors, apart from constant streaming of O_2_, H_2_O_2_, KBrO_3_, and (NH_4_)_2_S_2_O_8_ were added to the investigated suspension. In addition, to examine the influence of active species such as ^•^OH radicals and photogenerated holes, ethanol or NaI were added into the suspension.

### 2.3. Analytical Monitoring of Photodegradation 

To monitor the photodegradation of AMI, high pressure liquid chromatography with a diode array detector (UFLC-DAD, Shimadzu Nexera, Tokyo, Japan) was used. Aliquots of the reaction mixture were taken before the start of irradiation and at specific time intervals during the irradiation (volume variation ca. 10%). All samples with photocatalyst were filtered through a Millipore (Millex-GV, Burlington, MA, USA, 0.22 μm) membrane filter in order to separate the catalyst particles. Prepared aliquots were analyzed on UFLC-DAD as described previously [20]. pH was measured using a combined glass electrode (pH-Electrode SenTix 20, WTW, Thermo Fisher Scientific, Waltham, MA, USA) connected to a pH meter (pH/Cond 340i, WTW). Samples for measurements of temporal changes in the total organic carbon (TOC) were irradiated at different time intervals and analyzed after filtration on an Elementar Liqui TOC II analyzer (Elementar, Langenselbold, Germany) according to Standard US EPA Method 9060A. Ion chromatography (IC) analysis was performed on a Dionex ICS 3000 Reagent-Free IC system (Thermo Scientific, Carlsbad, CA, USA) with a conductometric detector [21].

### 2.4. Toxicity

Assessment of the cytotoxic effect on the growth of mammalian cell lines was performed, similar to our previous investigations [22] with the difference being that in this work, the concentration of AMI was 0.0300 mmol/L and photocatalyst loading was 1.0 mg/mL. Aliquots of 2 mL suspension of AMI were taken at the beginning of the experiment, as well as at different times during the irradiation, and after that were filtered through 0.22 μm membrane filters (Sartorius, Goettingen, Germany). The cell lines were grown in DMEM medium, supplemented with 10% heat inactivated FCS, 100 IU/mL of penicillin, 100 μg/mL of streptomycin, and 0.25 μg/mL of amphotericin B. Cells were cultured in 25 mL flasks (Corning, New York, NY, USA) at 37 °C in the atmosphere of 5% CO_2_ and high humidity, and sub-cultured twice a week. A single cell suspension was obtained using 0.1% trypsin with 0.04% EDTA.

Reaction mixtures of AMI and ZnO (20 μL) were added to 180 μL of the culture medium with cells. The same volume (20 μL) of ultrapure water was added to the control wells. Thus, the final concentration of all substrates was 3 μmol/L. The blank tests were performed using pure AMI solution, as well as the aqueous suspension of ZnO (without substrate), which were sonicated in the dark for 15 min and filtered through 0.22 μm membrane filters. Evaluation of cell growth was done by the colorimetric SRB assay of [23], which was modified by Cetojevic-Simin et al. [24].

## 3. Results and Discussion

### 3.1. Photocatalysts Screening

**Figure 2:**
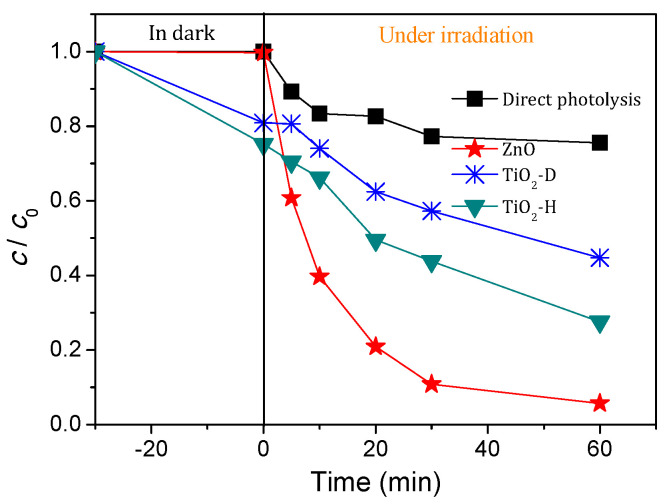
Kinetics of direct photolysis and photocatalytic degradation of AMI (0.0300 mmol/L) in the presence of different nanopowders (1.0 mg/mL) using solar radiation.

The efficiency of photocatalysts ZnO, TiO_2_-D, and TiO_2_-H in photocatalytic degradation of AMI under solar irradiation was investigated. As can be seen from Figure 2, photocatalytic degradation of AMI using ZnO proceeded faster than the other two photocatalysts, and after 60 min of irradiation, 94.3% of AMI was degraded. On the other hand, the degradation efficiency of AMI differed to a lesser extent in the presence of TiO_2_-D and TiO_2_-H, whereby the system with TiO_2_-H proved to be somewhat more efficient. Namely, in the presence of TiO_2_-D and TiO_2_-H, 55.3% and 72.4% of AMI was respectively degraded after 60 min of irradiation. The higher photocatalytic efficiency of AMI in the presence of ZnO could be attributed to the better mobility, generation, and separation of e^−^−h^+^ pairs of ZnO in comparison with TiO_2_ [25]. The blank experiments for either irradiated aqueous AMI solution (direct photolysis, Figure 2) or AMI solution in the dark showed that both the photocatalyst and irradiation are necessary for the removal of this antidepressant drug, mainly referring to the study of AMI stability in the dark, where it showed complete stability over a period longer than 850 days. In addition, Figure 2 shows the occurrence of adsorption of AMI in the dark, whereby the percentage of adsorption depended on the photocatalyst type. Higher adsorption of AMI (24.8%) was noticed with TiO_2_-H and after 30 min of sonification in the dark, as opposed to 19.1% of AMI with TiO_2_-D, while adsorption of AMI on the ZnO surface was not detected. Considering that the ZnO has been proven to be the most effective photocatalyst for the photodegradation of AMI, it was further used for all experiments.

### 3.2. Various Sources of Radiation

**Figure 3:**
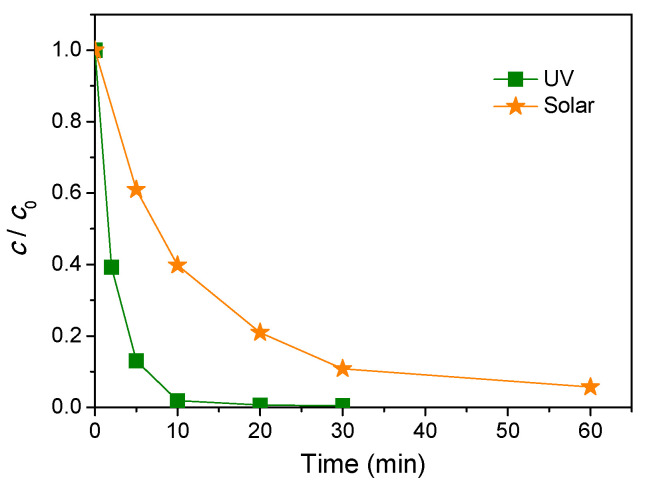
Effect of radiation type on the kinetics of AMI (0.0300 mmol/L) photocatalytic degradation in the presence of ZnO (1.0 mg/mL).

In order to investigate the influence of different types of radiation on the efficiency of AMI removal, an aqueous suspension of AMI was irradiated with UV or solar radiation. As shown in Figure 3, UV light led to the complete degradation of AMI after 30 min of irradiation, while after 60 min under solar radiation, 94.3% of AMI was removed. In Table 3 are presented the kinetics parameters, and it can be seen that in the case of UV, the degradation rate was 4.3 times higher than in the case of the system under solar radiation. These results could be explained by the fact that in the reaction system with solar radiation, a smaller number of photons from the UV-spectrum were present, and hence the formation of highly reactive species was smaller, and accordingly, the catalytic activity of ZnO under solar radiation was also smaller. Owing to the fact that the efficiency of degradation of AMI was high enough, all further measurements were performed using solar radiation.

### 3.3. Effects of ZnO Loadings

**Figure 4:**
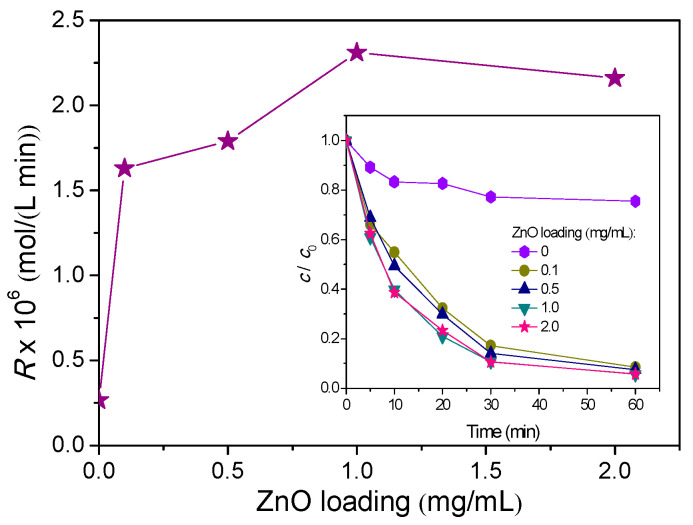
Effects of ZnO loadings on the photocatalytic degradation rate of AMI, calculated for the first 20 min of solar irradiation. Inset represents effects of ZnO loadings on the kinetics of photocatalytic degradation of AMI.

It is known that the photocatalytic degradation rate proportionally depends of the photocatalyst loading in such a way that with increased loading, degradation rate increases until the optimum value. Namely, loading increase has two opposite effects on the efficiency of the photocatalytic process. On one hand, its higher concentrations in the reaction mixture increase the number of active sites for adsorption of pollutants on the photocatalyst surface, which increases the efficiency of degradation. On the other hand, higher photocatalyst loading leads to aggregation of its particles, which increases the dispersion of light from the photocatalyst surface. In addition, aggregation leads to a reduction in the surface area. Therefore, the photocatalyst surface is not available for the generation of e^−^−h^+^ pairs, which can reduce the efficiency of photocatalytic degradation [26]. A series of experiments were carried out to investigate the effect of ZnO loadings on the efficiency of AMI photodegradation. ZnO loading was varied from 0.1 to 2.0 mg/mL (Figure 4, inset). Figure 4 represents the dependence of AMI photodegradation rate on the function of the ZnO loadings. Based on the obtained results, it can be observed that the degradation rate increases with increased photocatalyst loadings up until 1.0 mg/mL, and above this value the degradation rate slightly drops. Based on the above, it can be concluded that the optimal ZnO loading for the degradation of AMI was 1.0 mg/mL under solar radiation. Similar results were also obtained by other researchers [27,28].

### 3.4. Effect of Amitriptyline Concentration

**Figure 5:**
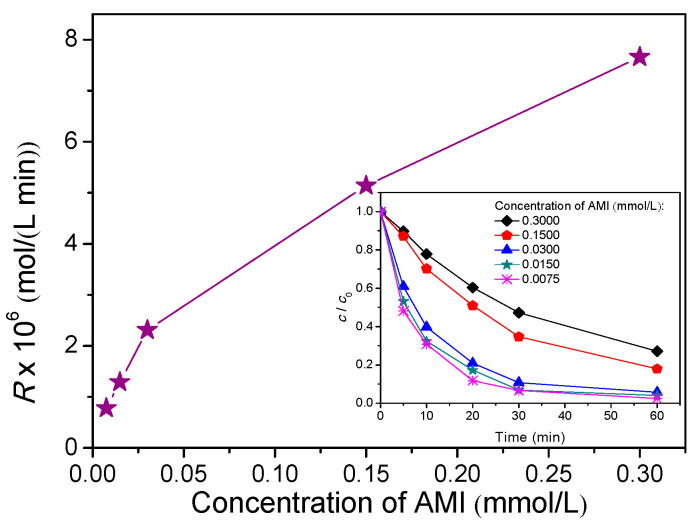
Effect of the initial AMI concentration on the photocatalytic degradation rate calculated for the first 20 min of irradiation in the presence of ZnO (1.0 mg/mL) and solar radiation. Inset represents the effect of initial AMI concentration on the kinetics of photocatalytic degradation.

The influence of various initial concentrations of AMI on the efficiency of photocatalytic degradation was investigated. Namely, AMI concentration was varied in the range of 0.0075 to 0.3000 mmol/L (Figure 5, inset). Figure 5 represents the photocatalytic degradation rate of AMI as the function of its initial concentrations, and the results revealed that the degradation rate increased with increases in AMI concentration. This phenomenon is probably due to the fact that in the system with no change in photocatalyst loadings and radiation intensity, with increasing of the substrate concentration to optimal value, the number of occupied sites on the photocatalyst is increased. This situation consequently increases the utilization of radiation, which in the end leads to an increase in the photocatalytic degradation rate of AMI, which is in accordance with literature data [29]. However, the increase in degradation rate is not exactly proportional to the increase of the AMI concentration, which can be seen from the appropriate degradation rates showed in Figure 5.

### 3.5. Effect of the Presence of Various Electron Acceptors

**Figure 6:**
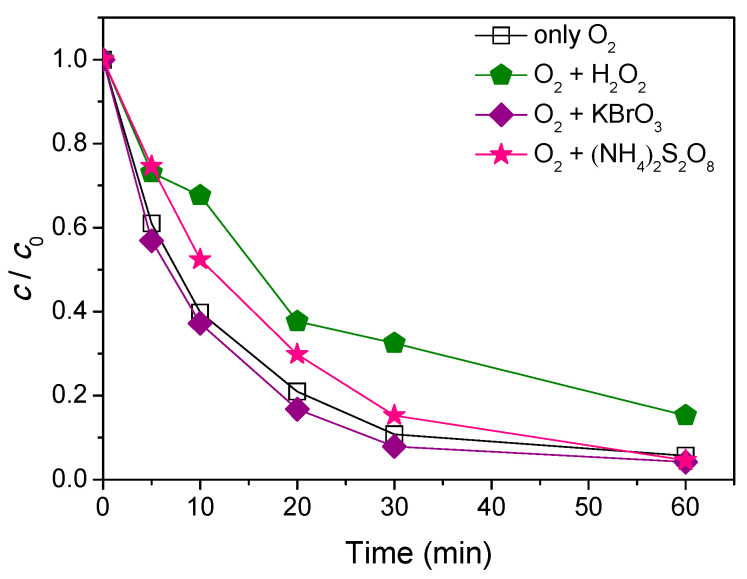
The effect of addition of various electron acceptors (3.0 mmol/L) on the kinetics of AMI (0.0300 mmol/L) photocatalytic degradation using ZnO (1.0 mg/mL) and solar radiation.

The recombination of e^−^−h^+^ pairs represents one of the biggest problems in the application of photocatalysts. This phenomenon is very pronounced in the absence of a suitable electron acceptor, potentially causing reduction in the efficiency of the photocatalytic process. In order to increase the formation of ^•^OH radicals and prevent recombination of e^−^−h^+^ in the reaction mixture, an electron acceptor was added. O_2_ is the most commonly used electron acceptor. Beside O_2_, KBrO_3_, H_2_O_2_, and (NH_4_)_2_S_2_O_8_ are usually used as electron acceptors [30]. Apart from the influence of O_2_, which was introduced in all cases, the influence of the presence of H_2_O_2_, KBrO_3_, and (NH_4_)_2_S_2_O_8_ on AMI degradation efficiency was tested (Figure 6).

As can be seen in Figure 6, the addition of H_2_O_2_ in the reaction systems reduced the efficiency of AMI degradation as compared with the systems in which only O_2_ was introduced. Such results can be a consequence of formation of peroxo radicals, which have a lower activity in the process of degradation as compared to the ^•^OH radicals [31]. Besides the addition of H_2_O_2_, (NH_4_)_2_S_2_O_8_ also reduced degradation efficiency of AMI compared to the systems with only O_2_ in the role of electron acceptor. However, this reduction in degradation efficiency was only observed during the first 30 min of the irradiation, while after 60 min it was the same as with O_2_. On the other hand, the presence of KBrO_3_ slightly elevated AMI’s degradation rate as compared to the systems that contained only O_2_, and after 60 min of irradiation, 95.8% of AMI was degraded. These results can be explained in a way that ${\mathrm{BrO}}_{3}^{-}$ ions react with the electrons from the conduction band of the photocatalyst, which reduces the possibility of recombining e^−^−h^+^ and therefore extends the lifetime of holes formed in the valence band [30]. Based on the obtained results, it can be concluded that the studied electron acceptors (H_2_O_2_, (NH_4_)_2_S_2_O_8_, and KBrO_3_) have different effects on the process of photocatalytic degradation of AMI. 

### 3.6. Active Species Identification

The most common reaction represented in photocatalytic degradation is that between the substrate and the adsorbed ^•^OH radicals on the surface of the photocatalyst or the reaction of direct charge transfer between the substrate and holes formed in the valence band of the photocatalyst [32]. To investigate the possible mechanism of photocatalytic degradation of AMI, the influence of active species such as ^•^OH radicals and photogenerated holes in the process of photocatalytic degradation was studied by addition of the specific scavengers.

**Figure 7:**
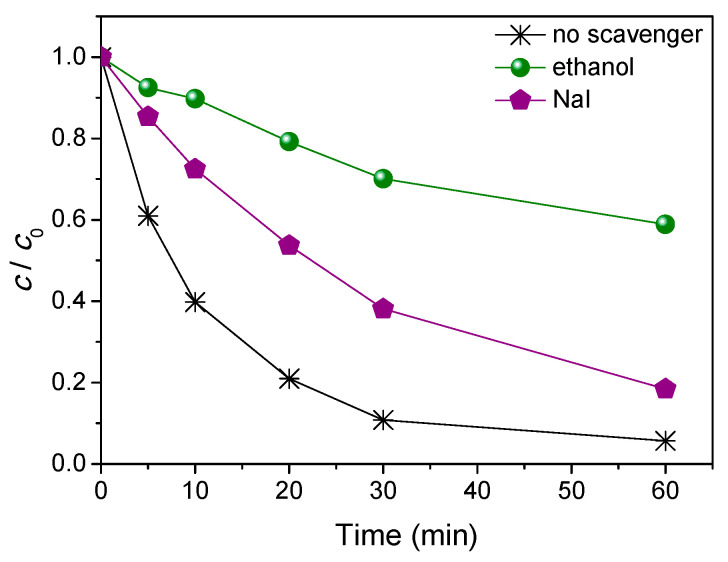
The effect of ethanol and NaI (3.0 mmol/L) on the kinetics of photocatalytic degradation of AMI (0.0300 mmol/L) using ZnO (1.0 mg/mL) and solar radiation.

The experiments were performed by the addition of ethanol and NaI in the reaction mixture. In fact, ethanol is mainly used as a scavenger of ^•^OH radicals [27], whereas the I^−^ ion is used as a scavenger of adsorbed ^•^OH radicals and photogenerated holes [32]. The obtained results (Figure 7) showed that the efficiency of AMI degradation was significantly reduced with the addition of ethanol into the reaction mixture in comparison with the results in its absence. After 60 min of irradiation in the presence of ethanol, only 41.1% of AMI was removed, whereas in the absence of ethanol, 94.3% of AMI was degraded. In addition, with the addition of NaI, the efficiency of photocatalytic degradation was reduced, whereas after 60 min of irradiation, 81.6% of AMI was removed. Based on these experimental results it can be concluded that the main photocatalytic degradation of AMI takes place via ^•^OH radicals free in solution, which is in agreement with other investigations [33,34,35], while the valence band holes have a secondary roles, as this was also expected from other research data [27,28,36]. 

### 3.7. Mineralization Studies of AMI

**Figure 8:**
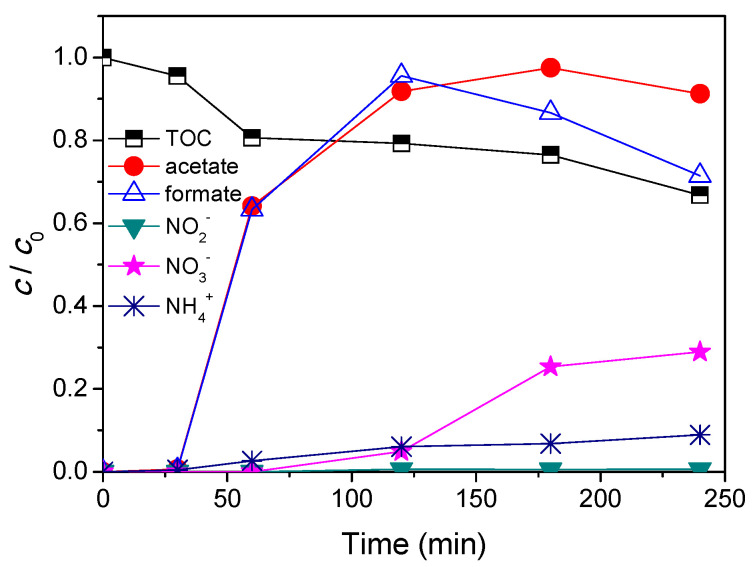
Degree of mineralization and evolution of different ions formed during AMI (0.0300 mmol/L) photocatalytic degradation in the presence of ZnO (1.0 mg/mL) and solar radiation.

For successful application of photocatalysis, the most important information is related to the degree of mineralization achieved during the process and the kinetics of disappearance of both the parent compound and by-products [5,8]. In order to assess the degree of mineralization during photocatalytic degradation of AMI, a decrease of the total organic carbon was estimated. In general, at low pollutant concentration or for compounds which do not form important intermediates, complete mineralization and reactant disappearance proceed with similar half-lives. However, at higher pollutant concentration where important intermediates occur, mineralization is slower than the degradation of the parent compound [37]. In addition, monitoring of ammonium, nitrate, nitrite, acetate, and formate ions by ion chromatography provides useful data for evaluating AMI degradation. The degree of AMI mineralization, as well as the evolution of different ions formed during photocatalytic degradation is shown in Figure 8. Based on the obtained results it can be seen that after 240 min of irradiation only 33.2% of AMI was mineralized. In addition, unsurprisingly it can be concluded that the mineralization and the evolution of ions were slower than the kinetics of AMI removal with ZnO (Figure 2). Beside CO_2_ and H_2_O, looking at the formula of AMI (Figure 1, inset), it can be expected that NH_4_^+^ and/or NO_3_^−^/NO_2_^−^ as well as acetate and formate can be formed during the photocatalytic degradation of AMI. Nitrogen-containing molecules are mineralized into NH_4_^+^ and mostly NO_3_^−^, and formed ammonium ions are relatively stable, and their proportions depend mainly on the initial oxidation of nitrogen and on the irradiation time. In addition, the concentrations of NH_4_^+^ and NO_3_^−^ increased with increasing irradiation time, while formation of NO_2_^−^ ions was negligible. Further, the concentration of acetate increased up until 180 min of irradiation and then decreased, while in the case of formate, a surge was observed up until 120 min, after which it decreased.

### 3.8. Toxicological Assessments of AMI and Formed Intermediates

Toxicity investigations are very important for environmental ecology, especially for the case of implementation of the catalyzed irradiation method. In order to evaluate the cytotoxicity of AMI, as well as the intermediates of the photocatalytic degradation with ZnO, in vitro growth of the four cell lines were investigated, namely H-4-II-E, Neuro-2a, HT-29, and MRC-5. 

**Figure 9:**
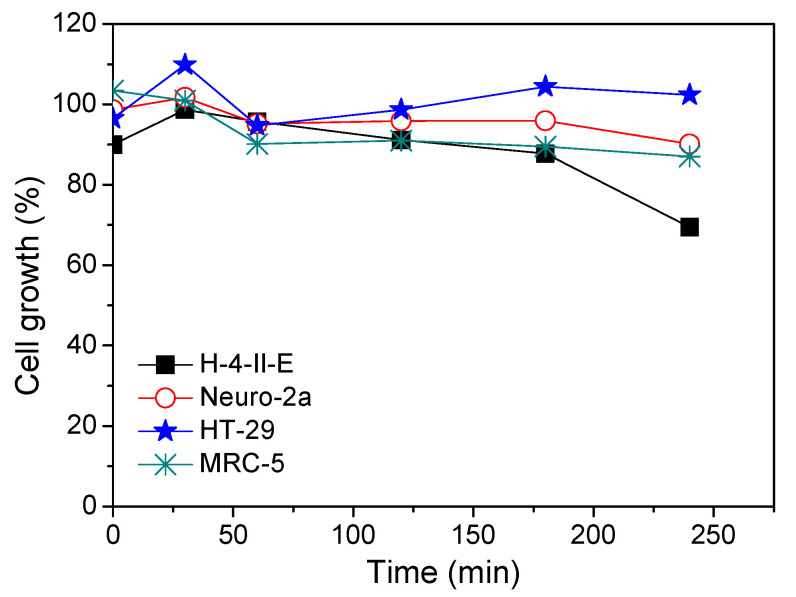
Cytotoxic activity of AMI (0.0300 mmol/L) and intermediates formed during photocatalytic degradation using ZnO (1.0 mg/mL) and solar radiation on the selected cell lines.

Based on the obtained results (Figure 9), it can be seen that the growth of selected cell lines depended on irradiation time (and intermediates formed during photocatalytic degradation) and type of cell lines. H-4-II-E cell lines were shown to be the most sensitive, with growth inhibition in the range of 1.34–30.6% inhibition, and highest values were found for the longest degradation time (240 min). Significant growth inhibition was observed for MRC-5 cell lines (from 9.0 to 12.9%), whereby mild stimulation of cell growth was obtained for samples at 0 min and after 30 min of irradiation (3.41 and 0.92%, respectively). This was probably due to the formation of cytotoxic intermediates after 60 min of irradiation. In Neuro-2a cells, growth was inhibited in the range from 1.31 to 9.84%, while only mild stimulation of cell growth (1.72%) was recorded after 30 min of irradiation. In the HT-29 cells, only a mild inhibition of growth was observed, ranging from 1.34 to 5.29%. Cell growth stimulation of HT-29 cells was from 2.32 to 9.83%, which was observed after 240 and 30 min of irradiation, respectively.

**Figure 10:**
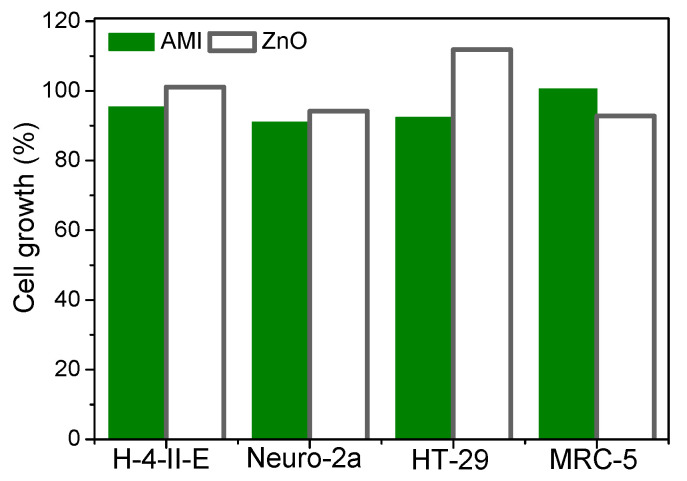
Cytotoxic activity of AMI (0.0300 mmol/L) and the blank probe ZnO (1.0 mg/mL) on the investigated cell lines.

In addition, the influence of AMI and the blank probe on cell growth was examined (Figure 10). It was found that AMI inhibited the growth of H-4-II-E, HT-29, and Neuro-2a cells (4.66, 7.60, and 8.91%, respectively). In MRC-5 cells, stimulation of growth was observed (0.53%). Results of a blank test (in the presence of ZnO, and absence of AMI) showed cell growth inhibition in Neuro-2a and MRC-5 cells (5.84 and 7.20%, respectively) and cell growth stimulation in H-4-II-E and HT-29 cells (1.14 and 11.8%, respectively). Although ZnO nanoparticles (NPs) have wide application, i.e., they are used in food products as additives and supplements, and in containers and packaging; in the energy sector as fuels and catalysts; in consumer electronics in semiconductors and air filtration; in pharmaceuticals; in biomedical engineering; and in drinking water, they may be toxic due to their partial dissolution. Thus, the concentrations of free Zn^2+^ in ZnO NP solutions needs to be measured to understand the effects of ion dissolution at different concentrations of the particles, which is important for defining toxicity. Hence, more investigation, with standard experimental conditions, is needed to better understand ZnO NP toxicity at the cellular and physiological levels, as these NPs may enter the food chains. Environmental and human exposure due to nanomaterial residues in water, air, soil, and crops is expected to increase with exposure routes, including possible bioaccumulation in the environment and food chain [38,39,40,41].

Finally, based on the obtained results, none of the samples produced cell growth inhibition higher than 50% (Figure 9 and Figure 10), and their toxicity was substantially lower compared to the cytotoxic drugs and the known toxicant, HgCl_2_ [42].

## 4. Conclusions

The results of this study indicate that ZnO can efficiently catalyze the photodecomposition of AMI in the presence of solar light and oxygen. The results also revealed that the degradation rate is influenced by the different parameters, such as catalyst loading, initial substrate concentration, presence/absence of various electron acceptors in addition to molecular oxygen, as well as ^•^OH radicals and hole scavengers. The photocatalytic degradation of AMI in aqueous TiO_2_ suspensions in the first period of degradation follows pseudo first-order kinetics. The experimentally determined optimum loading of the photocatalyst was 1.0 mg/mL. Moreover, the degradation rate increased with the increase of AMI initial concentration. The influence of H_2_O_2_ and (NH_4_)_2_S_2_O_8_ as electron acceptors in the first period decreased the reaction rate, while KBrO_3_ slightly increased the degradation rate of AMI. It was also found that the presence of ethanol as a scavenger of ^•^OH radicals inhibited the AMI photodecomposition more than NaI, suggesting that the reaction mechanism mainly involves free ^•^OH radicals, and partially holes. The disappearance of AMI was accompanied by the release of ionic intermediates and byproducts, namely NH_4_^+^, NO_3_^−^, NO_2_^−^ acetate, and formate, while the mineralization degree was ~30% after 180 min of irradiation. Cell growth in the majority of cell lines was mildly affected by the mixture of AMI and the respective photocatalytic degradation intermediates obtained using ZnO catalyst and solar irradiation, while at the longest irradiation time, it was moderately affected in the most sensitive cell line. The results of these investigations clearly demonstrated the importance of choosing the optimum degradation parameters to obtain high substrate degradation efficiency and low toxicity, which are essential for any practical application of photocatalytic oxidation processes.

## Figures and Tables

**Table 1:** Properties of AMI.

Therapeutic Group	Tricyclic Antidepressant
Molecular formula	C_20_H_24_ClN
Molecular weight (g/mol)	313.9
p*K*_a_	9.4 ^a^

^a^ Data extracted from [16]

**Table 2:** Operational variables applied during photocatalytic degradation of AMI.

Process Variable	Unit	Value
Suspension volume	mL	20
Average light intensity, UV	mW/cm^2^	5.304
Average light intensity, solar	mW/cm^2^	63.85 (Vis region); 0.219 (UV region)
Initial AMI concentration	mmol/L	0.0075–0.3000
Catalyst loading	mg/mL	0.1–2.0
pH	-	~6.7 (ZnO); ~5.0 (TiO_2_-D); ~4.9 (TiO_2_-H)
O_2_ flow	mL/min	3.0
Sonification intensity	Hz	50
Sonification duration	min	30
Temperature	°C	25
Electron acceptor concentration	mmol/L	3.0
^•^OH radical and hole scavengers concentration	mmol/L	3.0

**Table 3:** Effect of radiation type on AMI degradation rate (0.0300 mmol/L) in the presence of ZnO (1.0 mg/mL).

Type of Radiation	*R*^a^ × 10^6^ (mol/(L min)) ^a^	*r* ^b^
UV	11.8	0.998
Solar	2.76	0.999

*R*^a^—Degradation rate determined for the first 10 min of irradiation; *r*^b^—linear regression coefficient.

## Data Availability

Not applicable.
